# Neurobiological insights from bioluminescence imaging

**DOI:** 10.18632/oncotarget.20302

**Published:** 2017-08-13

**Authors:** Markus Aswendt, Franziska Melanie Collmann, Mathias Hoehn

**Affiliations:** Mathias Hoehn: In-vivo-NMR Laboratory, Max Planck Institute for Metabolism Research, Cologne, Germany and Department of Radiology, Leiden University Medical Center, Leiden, The Netherlands

**Keywords:** bioluminescence imaging, brain, stroke, neurogenesis, inflammatory response

Bioluminescence imaging has already been used for two decades to visualize cells and target genes completely non-invasively. Most frequently, firefly luciferase is applied which efficiently converts the substrate D-Luciferin in an enzymatic reaction that is dependent on ATP, Mg^2+^ and O_2_. Commercial imaging equipment is available to capture the emitted photons when luciferase is expressed in cells of a transgenic animal or in grafted transgenic cells with high efficiency. While the first *in vivo* applications were restricted to tumor cells, cell tracking was expanded to stem cells and to target more specifically gene expression, infection and protein-protein interactions [1]. The substrate D-Luciferin needs to be injected into the animal before every measurement, however, it is non-toxic and crosses the blood-brain barrier, which makes bioluminescence also attractive for neuroscience. Compared to subcutaneous tumor growth measures with bioluminescence imaging, the brain appears to be a much more difficult organ to be explored with light. Indeed, bioluminescence is dependent on the physical constraints of light scattering and absorption in deep tissues. However, in contrast to fluorescence imaging, there is less background signal, as the photons are only emitted by luciferase expressing cells and the light needs to penetrate the tissue only once [2]. We started to evaluate bioluminescence imaging for tracking neural stem cells in animal models of neurological disorders by comparing different imaging reporters such as luciferases isolated from different species, and defined an optimal imaging protocol to overcome the limitations of applying bioluminescence in the mouse brain. We could show that in a comparison of luciferases from the firefly, sea pansy and click beetle only the firefly luciferase Luc2, which is codon-optimized for expression in mammalian cells and emits with high quantum yield at 610 nm *in vivo*, is optimal for the mouse brain application. Furthermore, luciferases utilizing different substrates such as firefly and renilla, as well as green- and red-shifted variants for spectral unmixing [3] can be combined in a dual reporter approach. Through an extensive evaluation of the imaging protocol parameter, such as anaesthesia, the type of substrate injection, substrate concentration and timing, we achieved a signal gain of 200% compared to previous protocols and reliably detected 1,500 neural stem cells [4,5]. The temporal differentiation profile of neural stem cells transplanted into mouse brain cortex was thus unravelled by following the bioluminescence imaging intensity with luciferase expression set under specific gene control reflecting early and late neuronal maturation [6].

In our most recent study we used bioluminescence imaging to map the age-dependent longitudinal effect of stroke on neurogenesis [7]. Stroke is a leading cause of death and disability worldwide and as there are no treatments available beyond the acute phase, one promising approach is to explore the potential of endogenous repair processes such as neurogenesis. We imaged three age groups, 2, 6, and 12 months old transgenic mice, in which luciferase is selectively expressed in doublecortin-positive neuronal progenitor cells, and induced experimental stroke with the middle cerebral artery occlusion model. Bioluminescence imaging provided unique insight into the temporal profile of neurogenesis: the maximal upregulation occurred at 4 days post stroke followed by a continuous decrease to basal levels by three weeks post stroke. Older animals effectively compensated for reduced levels of basal neurogenesis by an enhanced sensitivity to the cerebral lesion, resulting in upregulated neurogenesis levels approaching those measured in young mice. In middle aged and older mice, but not in the youngest ones, additional upregulation of neurogenesis was observed in the contralateral healthy hemisphere. While conventional histological quantifications would have missed the individual temporal relationship of pre- and post-stroke neurogenesis, bioluminescence imaging was here key to identify sustained potential of neurogenesis to contribute to brain repair.

Future activity will exploit this sensitive non-invasive imaging modality to decipher the temporal dynamics of the inflammatory response after stroke. The switch between pro-inflammatory and protective phenotypes of macrophages and microglia can be monitored using cell-specific and phenotype-specific luciferase signal [8], thus providing essential information for the understanding of the interaction between stem cells and inflammatory cells, and finally contributing to novel therapeutical strategies based on these mechanisms.
